# Food Safety and Baby Food Handling Knowledge and Associated Factors Among Pregnant Women in Bangladesh: Findings From a Questionnaire‐Based Cross‐Sectional Survey

**DOI:** 10.1002/hsr2.72713

**Published:** 2026-06-30

**Authors:** Nitai Roy, Sultan Mahmud Imran, Aysha Siddiky, Abdullah Al Adib, Md. Mahmud Hasan, A. M. Jubayer, Jasmin Ara Farhana, Kamal Krishna Biswas

**Affiliations:** ^1^ Department of Biochemistry and Molecular Biology Patuakhali Science and Technology University Patuakhali Bangladesh; ^2^ Department of Community Health and Hygiene Patuakhali Science and Technology University Patuakhali Bangladesh; ^3^ Faculty of Nutrition and Food Science Patuakhali Science and Technology University Patuakhali Bangladesh; ^4^ Department of Human Nutrition and Dietetics Patuakhali Science and Technology University Patuakhali Bangladesh; ^5^ Department of Biochemistry and Molecular Biology University of Rajshahi Rajshahi Bangladesh

**Keywords:** Bangladesh, food handling, foodborne illness, infant formula, pregnancy

## Abstract

**Background and Aims:**

Pregnant women are particularly vulnerable to foodborne illnesses, which can have detrimental effects on both maternal and fetal health. This study aimed to assess the food safety and baby food‐handling knowledge among pregnant women in Bangladesh, along with associated risk factors.

**Methods:**

This cross‐sectional study was conducted among 341 pregnant women using a structured questionnaire from January to February 2024. Data were collected on sociodemographic characteristics, food safety knowledge across four domains (including personal and kitchen hygiene, symptoms of foodborne diseases, high‐risk foods and cross‐contamination, and storage and leftover food handling), and baby food handling knowledge. Multiple linear regression analysis was used to identify predictors of knowledge levels.

**Results:**

Participants demonstrated an overall food safety knowledge score with a mean score of 15.88 ± 6.07 out of 30 (52.9% correct), but significant gaps existed in areas such as cross‐contamination prevention, refrigeration temperatures, and thawing techniques. Baby food handling knowledge was slightly better with a mean score of 9.13 ± 3.86 (60.9% correct), although gaps existed regarding perishable food storage. The regression analysis revealed that older participants aged (≥ 25 years), those with no formal education, a family monthly income between 15000 and 30000 BDT, residing in rural areas, and in their first trimester of pregnancy had significantly lower food safety and baby food‐handling knowledge. Additionally, baby food‐handling knowledge was significantly lower among participants who experienced a second or subsequent pregnancy.

**Conclusion:**

This study highlights the need for targeted food safety educational interventions during antenatal care, particularly for older, uneducated rural pregnant women in their first trimester of pregnancy, to improve maternal and fetal health, as well as the health of newborns.

## 1 Introduction

Foodborne illnesses remain a global public health issue affecting millions of people each year. In 2019, the Centers for Disease Control and Prevention (CDC) reports approximately 9.9 million cases of domestically acquired foodborne illnesses in the United States, leading to 53,300 hospitalizations and 931 deaths [1]. Globally, the World Health Organization (WHO) estimates over 600 million cases and 420,000 fatalities each year due to food contamination [2]. Among these, diarrheal diseases are particularly severe, contributing to 550 million illnesses and 230,000 fatalities worldwide, with children being especially vulnerable, accounting for 220 million cases and 96,000 fatalities occurring in this age group annually [3]. However, the WHO South‐East Asia Region bears the highest burden, with more than 150 million cases, 175,000 deaths, and 12 million disability‐adjusted life‐years (DALYs) lost each year [4]. Additionally, in this region, 60 million children under the age of five suffer from foodborne diseases each year, resulting in 50,000 deaths [3]. In Bangladesh, foodborne diseases are a major concern, with approximately 30 million cases reported annually, including diarrheal infections, enteric fever, and hepatitis [5, 6]. These illnesses can lead to short‐term symptoms, such as food poisoning, and long‐term conditions, such as cancer, kidney or liver failure, and brain and neurological abnormalities, ultimately resulting in death, disability, significant economic losses, and developmental issues in infants [3, 7].

Food safety, as defined by the United States Department of Agriculture (USDA), involves preserving the quality and safety characteristics of food to reduce the risk of contamination and foodborne diseases [8]. Although food safety is a concern for everyone, it is particularly critical for pregnant women, their unborn children, and children under 5 years old. During pregnancy, the immune system becomes less effective at combating foodborne illnesses, and the developing immune systems of fetuses are particularly vulnerable to foodborne infections caused by pathogens such as *Listeria monocytogenes* and *Toxoplasma gondii* [9]. These pathogens can quickly cross the placenta and bind cellular receptors in the placenta, causing severe illnesses in both the mother and the fetus [10, 11]. Maternal listeriosis can lead to serious complications such as amnionitis, preterm labor, spontaneous abortion, stillbirth, and neonatal meningitis [12, 13]. Similarly, toxoplasmosis can result in eye or brain damage in unborn babies [14]. Infants are more susceptible to foodborne illness than adults because of their immature immune systems, lower body weight, lower stomach acid production, and lack of autonomy over meal preparation. Since mothers are often the primary caregivers for babies at home, their food safety practices may put their babies at risk for food‐borne illness [15].

Research shows that pregnant women are highly motivated to learn about health‐related topics because of concerns for both their own and their babies' well‐being [10]. However, there are gaps in knowledge about personal hygiene, cooked food storage, food handling at home, and the risks associated with consuming high‐risk foods [11, 16, 17, 18, 19]. For example, a Canadian study found inadequate knowledge about infant formula handling, and contamination risks from environmental factors, such as contact with animals or surfaces [20]. Many formula‐feeding mothers lacked instruction on formula preparation or storage, neglected safe‐use directions on formula packaging labels, failed to wash hands before preparation, did not clean bottle nipples between uses, and did not discard formula left standing for over 2 h [21].

Although several studies have examined food safety knowledge and practices among pregnant women in countries such as Ghana and Iran, and among mothers of young children in other low‐ and middle‐income settings [16, 22, 23], no published work in Bangladesh has focused specifically on pregnant women despite clear evidence of poor food safety KAP among general household handlers, rural women and childbearing mothers [24, 25, 26]. Existing Bangladeshi studies either target household food handlers, students, consumers or mothers in general, and typically measure only general food safety domains (hygiene, storage, cross‐contamination), without addressing pregnancy or infant feeding as distinct risk contexts [24, 25, 26]. Likewise, international research on infant formula and baby‐food safety has documented frequent errors in preparation, storage, and leftover handling among caregivers [21, 27], yet there is almost no quantitative work that integrates both general food safety knowledge and baby food/formula handling within a single instrument in low‐resource South Asian settings. Furthermore, while multiple Bangladeshi studies show that education, income, residence and other socio‐demographic factors strongly shape food safety knowledge and practices among household handlers and consumers [24, 25, 28], none analyze how these factors relate specifically to pregnant women's combined food safety and baby‐food handling knowledge in high‐poverty rural districts such as those in northern Bangladesh. This leaves a critical evidence gap in Bangladeshi pregnant women where unsafe household food handling directly threatens both maternal and infant health.

Several studies have assessed food safety knowledge, attitudes, and practices among pregnant women in countries such as Slovenia [11], UAE [18], Turkey [17], and Ghana [16]. However, no research has been conducted in Bangladesh on this particular group. Therefore, this study aimed to assess food safety and baby food handling knowledge and the factors influencing these behaviors in Northern Bangladesh. These findings will help promote the health and well‐being of Bangladeshi pregnant women by encouraging healthcare professionals and nutritionists to conduct educational sessions on safe food‐handling practices. Additionally, collaboration between the government and educational institutions could integrate food safety information into curricula, while engaging policymakers and stakeholders to improve practices and regulations.

## 2 Materials and Methods

### 2.1 Study Design and Setting

This cross‐sectional study was conducted in January and February 2024 in Kurigram District, located in Northern Bangladesh within the Rangpur Division. Kurigram was selected for this study based on convenience, given its accessibility, and the researchers' logistical feasibility of data collection. Kurigram is one of the most economically disadvantaged districts in Bangladesh, with high poverty rates and limited access to healthcare and proper nutrition [29, 30, 31, 32]. These socioeconomic factors make the population more vulnerable to foodborne illnesses due to challenges in maintaining food safety standards, such as inadequate refrigeration, improper food storage, and limited awareness of hygiene practices [33]. According to the 2022 Census of Bangladesh, the district consists of nine upazilas (sub‐districts) and 72 unions (smallest administrative unit), with a population of 2,329,161, spread across 605,724 households. The population density is 1,037 people per km^2^ and the literacy rate (age 7 and over) is 65.0% [34, 35]. The study population was drawn from two Upazilla health complexes (Ulipur and Kathalbari) and selected from households in four unions (Durgapur, Fulbari, Umar Majid, and Matrimongol). These locations were chosen to represent both healthcare facility‐based and community‐based populations, ensuring a diverse sample that reflected the district's socioeconomic and demographic variations.

### 2.2 Eligibility Criteria

The study included healthy pregnant women aged 18 years or older who were permanent residents of the study districts and of Bangladeshi nationality. The participants were required to provide informed consent, and have no physical or cognitive impairments that would hinder their participation. Pregnant women who were seriously ill at the time of data collection, unable to communicate effectively, or unwilling to participate were excluded from the study.

### 2.3 Sample Size and Sampling

The sample size was determined using Cochran's formula (*n* = z^2^p (1 − q)/d^2^) based on the assumption of a 95% confidence interval, 50% prevalence, and 5% margin of error [36, 37]. A sample size of 384 participants was calculated. However, due to various constraints such as lack of resources, unwillingness of participants, time constraints, and logistical challenges, the final sample size obtained was 341. With this sample size, the margin of error slightly increased to approximately 5.3%, while maintaining a 95% confidence interval. This prevalence of 50% was assumed as a conservative estimate due to the lack of prior data on food safety and baby food handling knowledge among pregnant women in the study setting. Although sample size was based on estimating proportions, mean knowledge scores were also analyzed as secondary continuous outcomes to explore associated factors.

This study employed convenience sampling, and selected participants based on accessibility and willingness to participate, which is commonly used in exploratory studies conducted in resource‐limited settings where a complete sampling frame is not available. At the selected Upazilla Health Complexes (Ulipur and Kathalbari), pregnant women attending antenatal care services during the data collection period were approached consecutively and invited to participate if they met the eligibility criteria. For the community‐based component, households within the selected unions (Durgapur, Fulbari, Umar Majid, and Matrimongol) were identified with the assistance of local health workers and community representatives. Eligible pregnant women residing in these households were approached based on their availability and willingness to participate. This combined facility‐ and community‐based sampling approach was used to capture a more diverse sample of pregnant women across different socio‐demographic backgrounds.

### 2.4 Data Collection Instruments and Procedures

The questionnaire was developed based on a thorough review of the existing literature on food safety knowledge among pregnant women, and was adapted from previously used and widely reported instruments in similar populations, ensuring that all relevant aspects were covered [10, 11, 16, 17, 19, 36, 38, 39, 40, 41]. Data were collected through face‐to‐face interviews, using a structured questionnaire. To ensure consistency in data collection techniques, five data collectors were carefully selected and provided with comprehensive training in a virtual classroom. The training covered all sections of the questionnaire, interviewing techniques, and ethical considerations to ensure that each collector was well equipped to gather accurate and reliable data. For pregnant women visiting the Upazilla Health Complex, interviews were conducted in the waiting room to minimize disruption and ensure convenience, with efforts made to maintain privacy and confidentiality during the interview process by maintaining distance from other patients and ensuring that responses were not shared or overheard by others. In the case of household interviews, participants visited their homes during their preferred time to accommodate their schedules. It took 15–20 min to complete, allowing participants to answer all questions thoroughly. To minimize missing data, completed questionnaires were reviewed on‐site by data collectors immediately after each interview. Any incomplete or unclear responses were verified with participants at the time of data collection whenever possible.

### 2.5 Predictor Variables Predictor Variables

The study included several predictor variables to assess their impact on food safety and baby food handling knowledge among pregnant women. These variables were categorized as follows: age (18–24 years, 25–34 years, and 35 years or older), educational level (no formal education, primary, secondary, higher secondary, and honors/masters or above), religion (Islam and Hindu), residential status (rural and urban), employment status (unemployed or employed), family monthly income (less than 15,000 BDT, between 15,000 and 30,000 BDT, and more than 30,000 BDT; for reference [1] USD ≈ 110 BDT, and the minimum monthly wage in Bangladesh was approximately 8,000 BDT or ≈ 73 USD), planned pregnancy (yes or no), pregnancy number (first pregnancy and second pregnancy or above), and pregnancy trimester (first, second, and third trimester).

### 2.6 Determination of Food Safety and Baby Food Handling Knowledge

To assess the food safety and baby food‐handling knowledge of pregnant women, a total of 44 close‐ended questions were employed. A set of 29 questions evaluated participants' knowledge of food safety with responses categorized as “Yes,” “No,” or “Don't know.” This section covers essential topics, such as personal and kitchen hygiene, symptoms of foodborne diseases, high‐risk foods, cross contamination, storage and leftover food handling. The scoring system assigned 1 point for “Yes” responses, while “No” and “Don't know” responses received 0 points. However, for certain items that required a negative response to indicate correct knowledge the scoring was reversed: 1 point was attributed for “No” responses, and 0 points for “Yes” and “Don't know” responses. All items were assigned equal weight, as each question represented an independent and equally important aspect of food safety and baby food handling knowledge. This approach was used to generate an overall composite knowledge score based on correct response proportions rather than differential item weighting. Furthermore, the scoring system was not intended to measure psychometric weighting but to provide a composite knowledge index based on correct response proportions across domains. Additionally, 15 questions assessed pregnant women's knowledge of handling and preparing food for babies. In this section, the scoring system was straightforward: “Yes” responses were marked as 1 point, while “No” and “Don't know” responses received 0 points. The Cronbach's alpha values for food safety knowledge and baby food handling knowledge were 0.90 and 0.80, respectively, indicating excellent and good internal consistency for the respective scales.

### 2.7 Validity

A rigorous process was followed to ensure the validity of the questionnaire. Initially, two skilled research assistants reviewed the content of the questionnaire to verify that it accurately covered the intended topic. The questionnaire was then translated from English to Bengali by a bilingual professional to maintain its accuracy and consistency. To further validate the translation, a back translation was conducted by another expert fluent in both languages. A pilot study was conducted with 20 pregnant women, who were not included in the main study. Based on the feedback from the pilot study, appropriate corrections and adjustments were made to refine the questionnaire. For example, the item “Raw food cause food poisoning” was modified to “Raw, undercooked, or improperly washed food is a high‐risk food for causing food poisoning” to better reflect the actual risk factors and improve accuracy following a pilot study.

### 2.8 Data Analysis

Data were analyzed using IBM SPSS Statistics, version 27.1.0 (IBM Corp., Armonk, NY, USA). Prior to analysis, data were checked for completeness and internal consistency; cases with missing or incomplete data were excluded from the final analysis. As the proportion of missing data was minimal, no imputation methods were applied. Descriptive statistics (frequencies and percentages for categorical variables; means and standard deviations for continuous variables, as appropriate) were computed to summarize the data. The distribution of continuous dependent variables was assessed using tests of normality (Shapiro–Wilk test) and inspection of histograms [42, 43]. Because the data exhibited a skewed distribution, non‐parametric tests were used for group comparisons. The Kruskal–Wallis H test was applied to compare three or more independent groups, and the Mann–Whitney *U* test was used for comparisons between two independent groups [44, 45]. Multiple linear regression models were fitted to explore the associations between food safety and baby food‐handling knowledge scores (dependent variables) and selected demographic characteristics (independent variables). All independent variables were entered simultaneously using a forced‐entry (enter) method; no stepwise or other automated variable selection procedures were used. This strategy was chosen to estimate the independent association of each predictor while adjusting for potential confounding, rather than to develop a data‐driven predictive model. After model fitting, standard assumptions of linear regression (linearity, homoscedasticity, independence, and normality of residuals) were examined to ensure validity [46, 47, 48]. All hypothesis tests were two‐sided, with a priori statistical significance set at *p* < 0.05. Effect sizes were expressed as regression coefficients (β) with corresponding 95% confidence intervals.

### 2.9 Ethical Consideration

The study protocol was reviewed and approved by the Institutional Ethical Committee of Patuakhali Science and Technology University (approval PSTU/IEC/2023/65 (13)). Prior to the interviews, written informed consent was obtained from participants who could read and write, whereas oral consent was obtained from those who could not. Participants were thoroughly informed during the introduction that the collected information would be used solely for research purposes and analyzed anonymously to ensure confidentiality.

## 3 Results

### 3.1 Characteristics of the Respondents

**Figure 1:**
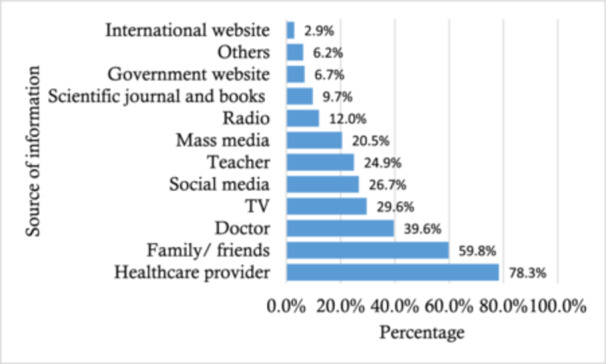
Source of information.

Due to logistical constraints, including limited resources, participant refusal, and time limitations, the final number of participants included in the study was 341. A total of 49 out of 390 eligible pregnant women declined to participate, resulting in a non‐participation rate of 12.6%. The primary reasons for non‐participation included lack of time and unwillingness to participate. Of the 341 participants, more than half (57.8%) were aged between 25 and 34 years old. Only 20.5% of the study participants had completed an honors/master's degree or higher, and the majority (75.1%) resided in rural areas. Approximately 83.0% of the participants were unemployed, and 36.1% reported a family monthly income below 15,000 BDT. Most participants (89.4%) planned their pregnancies, with 41.3% experiencing their first pregnancy. Nearly half (49.6%) of the women were in their 2nd trimester (Table 1). Participants primarily obtained information on food safety from healthcare providers and family/friends (Figure 1).

**Table 1:** Socio‐demographic characteristics of pregnant women (*n* = 341).

Variables	Frequency	Percentage (%)
**Age group (Years)**		
18–24	96	28.2%
25–34	197	57.8%
≥ 35	48	14.1%
**Education level**		
No Formal Education	40	11.7%
Primary	62	18.2%
Secondary	87	25.5%
Higher Secondary	82	24.0%
Honors/Masters or above	70	20.5%
**Religions**		
Islam	289	84.8%
Hindu	52	15.2%
**Residential status**		
Rural	256	75.1%
Urban	85	24.9%
**Employment status**		
Unemployed	283	83.0%
Employed	58	17.0%
**Family monthly income (BDT)**		
< 15,000	123	36.1%
15,000–30,000	151	44.3%
> 30,000	67	19.6%
**Planned Pregnancy**		
No	36	10.6%
Yes	305	89.4%
**Pregnancy number**		
First pregnancy	141	41.3%
Second pregnancy and above	200	58.7%
**Pregnancy trimester**		
First trimester	115	33.7%
Second trimester	169	49.6%
Third trimester	57	16.7%

Among the participants, 228 (66.9%) were recruited from Upazila Health Complexes and 113 (33.1%) were recruited through household visits (Supporting Information S1: Table S1). Comparisons between these two groups revealed several statistically significant differences. Women recruited through household visits were more likely to be older (≥ 35 years: 27.4% vs. 7.5%, *p* < 0.001) and to have lower educational attainment (no formal education: 29.2% vs. 3.1%, *p* < 0.001). They were also more likely to report higher parity (second pregnancy or above: 69.0% vs. 53.5%, *p* = 0.006) and to be in the first trimester at the time of interview (42.5% vs. 29.4%, *p* = 0.028). In contrast, participants recruited from health facilities tended to be younger and more educated.

### 3.2 Total Food Safety Knowledge

The overall mean food safety knowledge score among the participants was 15.88 ± 6.07, with a total correct response rate of 52.9% (Table 2).

**Table 2:** Food safety knowledge of pregnant women (*n* = 341).

Items (α = 0.90)	Responses		
	Yes (*n*, %)	No (*n*, %)	Don't Know (*n*, %)
**Personal and kitchen hygiene**			
It is important to wash hands in for at least 20 s before and after handling food.	299 (87.7)	3 (0.9)	39 (11.4)
It is important to wash hands after using toilets, changing diapers, using phone, and touching animals.	317 (93.0)	3 (0.9)	21 (6.2)
It is important to wash hands after handling raw meats or poultry.	284 (83.3)	13 (3.8)	44 (12.9)
It is important to wash hands after touching other body parts.	223 (65.4)	14 (4.1)	104 (30.5)
It is important to wash hands after sneezing.	232 (68.0)	24 (7.0)	85 (24.9)
It is important to wash hands after touching money.	165 (48.4)	36 (10.6)	140 (41.1)
It is important to use paper towels to clean up kitchen surfaces.	178 (52.2)	23 (6.7)	140 (41.1)
It is important to wash cutting boards, dishes, utensils, and countertops.	243 (71.2)	17 (5.0)	81 (23.8)
**Symptoms of food‐borne diseases**			
Diarrhea is a typical symptom of foodborne diseases like Toxoplasmosis.	309 (90.6)	6 (1.8)	26 (7.6)
Vomiting is a typical symptom of foodborne diseases like Listeriosis.	245 (71.8)	17 (5.0)	79 (23.2)
Nausea is a typical symptom of foodborne diseases.	176 (51.6)	29 (8.5)	136 (39.9)
Hypertension is a typical symptom of foodborne diseases.^a^	98 (28.7)	46 (13.5)	197 (57.8)
Hyperglycemia is a typical symptom of foodborne diseases.^a^	104 (30.5)	51 (15.0)	186 (54.5)
Coughing or sneezing is a typical symptom of foodborne diseases.^a^	73 (21.4)	114 (33.4)	154 (45.2)
**High risks foods and Cross‐contamination**			
Raw, undercooked, or improperly washed food is a high‐risk food for causing food poisoning.	291 (85.3)	17 (5.0)	33 (9.7)
Foodborne disease can be acquired from the consumption of contaminated food.	305 (89.4)	17 (5.0)	19 (5.6)
Storing raw and cooked food together causes contamination of food.	255 (74.8)	21 (6.2)	65 (19.1)
Sanitizer is good at killing bacteria.	207 (60.7)	29(8.5)	105(30.8)
Detergents are good at killing all viruses and bacteria.^a^	203 (59.5)	23 (6.7)	115 (33.7)
Use of gloves while handling food reduces the risk of food contamination.	184 (54.0)	31 (9.1)	126 (37.0)
Touching raw meats and then handling vegetables or other ready‐to‐eat foods can cause cross‐contamination.	184 (54.0)	23 (6.7)	134 (39.3)
Using separate cutting boards for meat and vegetables is helpful to avoid cross‐contamination.	194 (56.9)	26 (7.6)	134 (39.3)
The tasting of foods should be checked with a different spoon.	214 (62.8)	24 (7.0)	103 (30.2)
**Storage and leftover food handling**			
The temperature of the refrigerator should be less than or equal to 4°C.	147 (43.1)	4 (1.2)	190 (55.7)
Bacterial growth accelerates at a temperature of 75°C.^a^	59 (17.3)	14 (4.1)	268 (78.6)
Bacteria that cause food poisoning multiply rapidly at a temperature of 37°C.	65 (19.1)	12 (3.5)	264 (77.4)
Refrigerate or freeze meat, poultry, eggs, seafood, and other perishables within 2 h of cooking or purchasing.	119 (34.9)	14 (4.1)	208 (64.0)
Food thawed in cold water or in the microwave should be cooked immediately.	116 (34.0)	15 (4.4)	210 (61.6)
A large pot of food like soup, chili or stew should be divided into small portions and put in shallow containers before being refrigerated.	76 (22.3)	29 (8.5)	236 (69.2)
Refrigerated leftover food should be used within 2–3 days and cook it to proper temperatures.	140 (41.1)	20 (5.9)	181 (53.1)
**Total correct response (%)** b	52.9%		
**Total mean score** ± SD	15.88 ± 6.07		

^a^ Item was reversely coded.

^b^ Calculated by using this formula, Total correct response (%) $=\frac{\text{Total questions answered correctly}*100}{\text{Total numbers of questions}}.$

#### 3.2.1 “Personal and Kitchen Hygiene” Aspect

Most participants demonstrated a high level of knowledge of personal and kitchen hygiene (Table 2). The majority (87.7%) correctly identified the importance of washing hands for at least 20 s before and after handling food. Additionally, 93% acknowledged the need to wash their hands after using the toilet, changing diapers, using a phone, and touching animals. However, knowledge about hand hygiene practices varied, with only 48.4% recognizing that hands should be washed after handling money, and 52.2% agreeing on the importance of using paper towels for cleaning kitchen surfaces. The mean score for food safety knowledge related to personal and kitchen hygiene showed a significant association (all *p* < 0.05) with all demographic variables, except for religion and whether pregnancy was planned (Table 3).

**Table 3:** Relationship between demographic characteristics and food safety knowledge of pregnant women (*n* = 341).

Variables and categories	Personal and kitchen hygiene		Symptoms of food‐borne diseases		High risks foods and Cross‐contamination		Storage and leftover food handling	
	Mean ± SD	*p* value	Mean ± SD	*p* value	Mean ± SD	*p* value	Mean ± SD	*p* value
**Age groups (Years)**								
18–24	6.22 ± 2.17	**< 0.001**	2.96 ± 1.30	**0.006**	6.22 ± 2.07	**< 0.001**	1.48 ± 1.77	**0.008**
25–34	5.75 ± 2.16		2.81 ± 1.41		5.46 ± 2.10		2.18 ± 2.00	
≥ 35	4.35 ± 2.26		2.12 ± 1.63		3.79 ± 1.99		2.14 ± 1.84	
**Education level**								
No Formal Education	3.07 ± 1.70	**< 0.001**	1.37 ± 1.16	**< 0.001**	2.80 ± 1.30	**< 0.001**	1.27 ± 1.33	**< 0.001**
Primary	4.85 ± 2.42		2.50 ± 1.37		4.30 ± 2.19		0.62 ± 1.13	
Secondary	5.54 ± 2.17		2.49 ± 1.18		5.47 ± 2.08		1.26 ± 1.64	
Higher Secondary	6.52 ± 1.67		3.14 ± 1.27		6.31 ± 1.75		2.64 ± 1.69	
Honors/Masters or above	7.14 ± 1.83		3.65 ± 1.36		6.91 ± 1.20		3.71 ± 1.85	
**Employment status**								
Unemployed	5.44 ± 2.31	**< 0.001** a	2.61 ± 1.36	**< 0.001** a	5.2 ± 2.27	**< 0.001** a	1.66 ± 1.79	**< 0.001** a
Employed	6.89 ± 1.43		3.48 ± 1.59		6.60 ± 1.38		3.53 ± 1.86	
**Family monthly income (BDT)**								
< 15000	5.55 ± 2.46	**< 0.001**	2.78 ± 1.23	**< 0.001**	5.35 ± 2.29	0.090	1.14 ± 1.65	**< 0.001**
15000–30000	5.37 ± 2.14		2.47 ± 1.43		5.24 ± 2.30		2.07 ± 1.80	
> 30000	6.67 ± 1.78		3.35 ± 1.62		6.05 ± 1.70		3.32 ± 1.92	
**Religions**								
Islam	5.79 ± 2.16	0.138^a^	2.82 ± 1.40	0.088^a^	5.58 ± 2.15	**0.012** a	1.95 ± 1.94	0.320^a^
Hindu	5.13 ± 2.63		2.38 ± 1.57		4.67 ± 2.38		2.17 ± 1.92	
**Residential status**								
Rural	5.29 ± 2.32	**< 0.001** a	2.59 ± 1.39	**< 0.001** a	5.14 ± 2.27	**< 0.001** a	1.50 ± 1.66	**< 0.001** a
Urban	6.89 ± 1.48		3.24 ± 1.45		6.35 ± 1.69		3.42 ± 2.00	
**Planned Pregnancy**								
No	5.41 ± 1.84	0.208^a^	2.91 ± 1.59	0.463^a^	5.05 ± 2.04	0.170^a^	1.47 ± 1.57	0.155^a^
Yes	5.72 ± 2.29		2.74 ± 1.42		5.49 ± 2.22		2.04 ± 1.96	
**Pregnancy number**								
First pregnancy	6.07 ± 2.12	**0.009** a	2.90 ± 1.37	0.133^a^	5.99 ± 2.11	**< 0.001** a	2.05 ± 1.92	0.413^a^
Second pregnancy and above	5.42 ± 2.30		2.65 ± 1.47		5.06 ± 2.19		1.93 ± 1.95	
**Pregnancy trimester**								
First trimester	4.99 ± 2.16	**< 0.001**	2.61 ± 1.40	0.109	5.03 ± 2.18	**0.036**	1.96 ± 1.82	0.892
Second trimester	6.05 ± 2.18		2.78 ± 1.39		5.67 ± 2.18		2.01 ± 2.01	
Third trimester	6.03 ± 2.32		2.96 ± 1.61		5.59 ± 2.24		1.92 ± 1.96	
**Total**	**5.69** ± **2.25**		**2.75** ± **1.43**		**5.44** ± **2.20**		**1.98** ± **1.93**	

^a^ Mann‐Whitney *U* test was conducted, unless otherwise specified, and Kruskal‐Wallis H test was conducted. Bold values indicate statistical significance (*p* < 0.05).

#### 3.2.2 “Symptoms of Foodborne Diseases” Aspect

Awareness of common foodborne illness symptoms was varied among participants. While 90.6% correctly identified diarrhea as a symptom, only 51.6% recognized nausea as a potential sign. A significant number of respondents held misconceptions, with 28.7% and 30.5% mistakenly believing that hypertension and hyperglycemia, respectively, are symptoms of foodborne disease. Similarly, 21.4% of the participants incorrectly identified coughing or sneezing as typical symptoms. The mean score for food safety knowledge concerning symptoms of foodborne diseases demonstrated a significant relationship (all *p* < 0.05) with all demographic variables, except for religion, planned pregnancy, pregnancy number and pregnancy trimester. (Table 3)

#### 3.2.3 “High‐Risks Foods and Cross‐Contamination” Aspect

Knowledge of food contamination risks was relatively high, with 85% correctly identifying raw, undercooked, or improperly washed food as high‐risk factors for foodborne illnesses. Similarly, 89.4% of the participants understood that consuming contaminated food could lead to foodborne diseases. However, only 59.5% recognized the importance of using separate cutting boards for meat and vegetables, and 54% acknowledged the risk of cross‐contamination when touching raw meat and handling ready‐to‐eat food. The mean score of food safety knowledge regarding high‐risk foods and cross‐contamination showed a significant relationship (all *p* < 0.05) with all demographic variables except for family monthly income and planned pregnancy (Table 3).

#### 3.2.4 “Storage and Leftover Food Handling” Aspect

The participants' knowledge of food storage and leftover food handling was comparatively low. Only 43.1% correctly identified the recommended refrigerator temperature (≤ 4°C) and 34.9% knew that perishable foods should be refrigerated or frozen within 2 h. Misconceptions were prevalent, with 78.6% unsure about bacterial growth acceleration at 75°C (75°C was included to assess knowledge of proper food cooking temperatures, not bacterial growth acceleration) and 77.4% unaware that bacteria multiply rapidly at 37°C. The mean score of food safety knowledge regarding storage and leftover food handling symptoms showed a significant relationship (all *p* < 0.05) with age, educational knowledge, employment status, family monthly income, and residential status (Table 3).

### 3.3 Baby Food Handling Knowledge

The overall baby food handling knowledge score among the participants was 9.13 ± 3.86, with a total correct response rate of 60.9% (Table 4). Knowledge varied across items: while most women (83.9%) correctly recognized that dirty diapers should not be kept in the same bag as bottles or food, only 34.6% knew that frozen baby food can be stored for up to 3 months. Key gaps were noted in areas such as safe food storage duration (e.g., only 41.1% knew perishable items left unrefrigerated for over 2 h should be discarded) and honey avoidance (50.4% correct).

**Table 4:** Knowledge of pregnant women on handling and preparing food for babies (*n* = 341).

Items (*α* = 0.80)	Responses		
	Yes (*n*, %)	No (*n*, %)	Don't Know (*n*, %)
Detergent and hot water should be used to wash all blenders, food processors, and utensils that contact a baby's foods.	257 (75.4)	5 (1.5)	79 (23.2)
Bottles and food should be transported in an insulated cooler when traveling with the baby.	182 (53.4)	11 (3.2)	148 (43.4)
Milk, formula, or food left out of the refrigerator or without a cold source for more than 2 h should not be used.	140 (41.1)	22 (6.5)	179 (52.5)
You can store the frozen baby food for up to 3 months.	118 (34.6)	31 (9.1)	192 (56.3)
Formula can become contaminated during preparation.	225 (66.0)	26 (7.6)	90(26.4)
Preparing formula in smaller quantities on an as‐needed basis greatly reduces the possibility of contamination.	234 (68.6)	15 (4.4)	92 (27.0)
If using powder, reconstitution should be done immediately before feeding.	228 (66.9)	15(4.4)	98 (28.7)
If using liquid concentrates or ready‐to‐feed products, the label of instructions should be followed provided by the manufacturer.	228 (66.9)	21 (6.2)	92 (27.0)
Saliva on the spoon may contaminate the remaining food.	218 (63.9)	22 (6.5)	101 (29.6)
Honey should not be used as a sweetener to entice babies to drink water from a bottle.	172 (50.4)	23 (6.7)	146 (42.8)
Boiled water for at least 2 min before mixing it with formula.	168 (49.3)	15 (4.4)	158 (46.3)
Raw or unpasteurized milk or unpasteurized fruit or vegetable juice should not give to infants or young children.	222 (65.1)	15 (4.4)	104 (30.5)
Before preparing baby formula or bottled breast milk, hands should wash with soap and water.	266 (78.0)	10 (2.9)	65 (19.1)
Formula or bottled breast milk should not keep out at room temperature for more than 2 h.	172 (50.4)	21 (6.2)	148 (43.4)
Dirty diapers should not keep in the same bag as bottles or food.	286 (83.9)	13 (3.8)	42 (12.3)
**Total correct response (%)** ^**a**^	**60.9%**		
**Total mean score** ± SD	**9.13** ± **3.86**		

^a^ Calculated by using this formula, Total correct response (%) $=\frac{\text{Total questions answered correctly}*100}{\text{Total numbers of questions}}$

### 3.4 The Association Between Food Safety and Baby Food Handling Knowledge Scores and Characteristics of the Respondents

Table 5 presents the relationship between demographic characteristics, total food safety, and baby food‐handling knowledge scores of the respondents. Significant associations were found between most demographic variables (age, education, employment status, income, residential status, pregnancy number, and trimester) and food safety and baby food‐handling knowledge scores (all *p* < 0.05). However, no significant relationship was observed between religion, planned pregnancy and total knowledge of food safety.

**Table 5:** Relationship between demographic characteristics and total food safety and baby food handling knowledge score of pregnant women (*n* = 341).

Variables and categories	Food safety knowledge		Baby food handling knowledge	
	Mean ± SD	*p* value	Mean ± SD	*p* value
**Age groups (Years)**				
18–24	16.91 ± 5.43	**< 0.001**	9.71 ± 3.75	**< 0.001**
25–34	16.22 ± 6.07		9.44 ± 3.82	
≥ 35	12.41 ± 6.23		6.70 ± 3.40	
**Education level**				
No Formal Education	8.52 ± 3.43	**< 0.001**	4.62 ± 2.49	**< 0.001**
Primary	12.29 ± 5.26		7.12 ± 3.05	
Secondary	14.77 ± 5.11		8.58 ± 3.71	
Higher Secondary	18.63 ± 4.33		11.09 ± 3.05	
Honors/Masters or above	21.42 ± 3.39		11.88 ± 2.40	
**Employment status**				
Unemployed	14.93 ± 5.93	**< 0.001** a	8.61 ± 3.85	**< 0.001** a
Employed	20.51 ± 4.48		11.67 ± 2.80	
**Family monthly income (BDT)**				
< 15,000	14.84 ± 5.70	**< 0.001**	8.87 ± 3.81	**0.008**
15,000–30,000	15.15 ± 6.32		8.76 ± 4.17	
> 30,000	19.41 ± 4.84		10.46 ± 2.89	
**Religions**				
Islam	16.15 ± 5.91	0.075^a^	9.34 ± 3.80	**0.013** a
Hindu	14.36 ± 6.79		7.96 ± 4.05	
**Residential status**				
Rural	14.54 ± 5.85	**< 0.001** a	8.42 ± 3.77	**0** < **0.001** a
Urban	19.91 ± 4.84		11.29 ± 3.33	
**Planned Pregnancy**				
No	14.86 ± 5.22	0.220^a^	7.58 ± 2.56	**0.009** a
Yes	16.00 ± 6.16		9.32 ± 3.95	
**Pregnancy number**				
First pregnancy	17.03 ± 5.44	**0.004** a	9.70 ± 3.50	**0.019** a
Second pregnancy and above	15.07 ± 6.37		8.74 ± 4.06	
**Pregnancy trimester**				
First trimester	14.60 ± 5.82	**0.021**	8.40 ± 3.70	**0.038**
Second trimester	16.53 ± 6.11		9.62 ± 3.93	
Third trimester	16.52 ± 6.16		9.19 ± 3.83	

^a^ Mann‐Whitney *U* test was conducted, unless otherwise specified, and Kruskal‐Wallis H test was conducted. Bold values indicate statistical significance (*p* < 0.05).

Table 6 presents multiple linear regression models investigating factors associated with food safety and baby food‐handling knowledge among pregnant women. For food safety knowledge, the older age groups (25–34 years: *β* = −0.183, *p* < 0.001; ≥ 35 years: *β* = −0.186, *p* = 0.001) had significantly lower scores than those aged 18–24. Higher education levels showed a strong positive association, with the greatest knowledge among women with honors/master's degrees (*β* = 0.812, *p* < 0.001). Urban residents (*β* = 0.129, *p* = 0.006) and women in the later pregnancy trimesters (second: *β* = 0.147, *p* = 0.001; third: β = 0.198, *p* < 0.001) scored higher, while those with a monthly income of 15,000–30,000 BDT (*β* = −0.100, *p* = 0.036) had lower knowledge than the lowest income group (< 15,000 BDT).

**Table 6:** Multiple linear regression models investigating the factors associated with food safety and baby food handling knowledge of pregnant women (*n* = 341).

Variables	Food Safety knowledge				Baby food handling knowledge			
	*β*	95% CI		*p* value	*β*	95% CI		*p* value
		Lower Bound	Upper Bound			Lower Bound	Upper Bound	
**(Constant)**		7.112	11.955	**0.000**		3.032	6.354	**0.000**
**Age groups (Year)**								
18–24	***Ref.***				***Ref.***			
25–34	−0.183	−3.505	−0.991	**0.000**	−0.144	−1.992	−0.267	**0.010**
≥ 35	−0.186	−5.098	−1.388	**0.001**	−0.173	−3.199	−0.654	**0.003**
**Education level**								
No Formal Education	***Ref.***				***Ref.***			
Primary	0.159	0.666	4.348	**0.008**	0.145	0.187	2.713	**0.025**
Secondary	0.388	3.649	7.157	**0.000**	0.355	1.942	4.348	**0.000**
Higher Secondary	0.659	7.499	11.212	**0.000**	0.652	4.625	7.172	**0.000**
Honors/Masters or above	0.812	9.987	14.418	**0.000**	0.746	5.616	8.656	**0.000**
**Employment status**								
Unemployed	***Ref.***				***Ref.***			
Employed	0.034	−1.058	2.156	0.502	0.072	−0.364	1.841	0.189
**Family monthly income (BDT)**								
< 15,000	***Ref.***				***Ref.***			
15,000–30,000	−0.100	−2.355	−0.083	**0.036**	−0.158	−2.007	−0.448	**0.002**
> 30,000	−0.099	−3.431	0.398	0.120	−0.243	−3.673	−1.046	**0.000**
**Religions**								
Islam	***Ref.***				***Ref.***			
Hindu	−0.031	−1.798	0.757	0.423	−0.047	−1.379	0.374	0.260
**Residential status**								
Rural	***Ref.***				***Ref.***			
Urban	0.129	0.525	3.105	**0.006**	0.124	0.220	1.990	**0.015**
**Planned Pregnancy**								
No	***Ref.***				***Ref.***			
Yes	−0.007	−1.617	1.351	0.860	0.078	−0.034	2.002	0.058
**Pregnancy number**								
First pregnancy	***Ref.***				***Ref.***			
Second pregnancy and above	0.081	−0.191	2.184	0.100	0.108	0.033	1.663	**0.041**
**Pregnancy trimester**								
First trimester	***Ref.***				***Ref.***			
Second trimester	0.147	0.763	2.802	**0.001**	0.124	0.256	1.655	**0.008**
Third trimester	0.198	1.852	4.582	**0.000**	0.145	0.568	2.440	**0.002**

*Note:* CI = Confidence Interval; *β* = regression coefficient. The *R* ^2^ values for food safety, baby food‐handling and preparation knowledge were 0.548, 0.475, respectively. Bolded values indicate statistical significance (*p* < 0.05).

Similar trends were observed for baby food‐handling knowledge: older age (25–34: *β* = −0.144, *p* = 0.010; ≥ 35: *β* = −0.173, *p* = 0.003) and higher income (> 30,000 BDT: *β* = −0.243, *p* < 0.001) were negatively associated, while education (highest for honors/master's: *β* = 0.746, *p* < 0.001) and urban residence (*β* = 0.124, *p* = 0.015) had positive effects. Women in their second or subsequent pregnancies (*β* = 0.108, *p* = 0.041) and later trimesters (second trimester: *β* = 0.124, *p* = 0.008; third trimester: *β* = 0.145, *p* = 0.002) demonstrated better knowledge. Employment status, religion, and planned pregnancy were non‐significant predictors in both the models.

## 4 Discussion

This study assessed food safety and baby food‐handling knowledge and its associated factors among pregnant women in Northern Bangladesh. This study found an overall food safety knowledge score of 52.9% and a baby food handling knowledge score of 60.9% among participants. A recent study among meat handlers in Khulna city, Bangladesh, found that participants had a higher level of food safety knowledge (74.3%) [49], while another study on hospital service workers also reported relatively higher knowledge scores (59.7%) [50]. These variations suggest that occupational training, regulatory oversight, and access to food safety education may play critical roles in shaping knowledge. Interestingly, a study of university students in Bangladesh showed lower food safety knowledge (41.8%) [51]. This contrast highlights the potential differences in food safety knowledge between pregnant women and university students, implying that pregnancy may serve as a catalyst for improved food safety knowledge. The total food safety knowledge level in the current study was closely aligned with the findings of another study conducted among pregnant women in the UAE (53.4%) [38], while outperforming Jordanian pregnant women (46.6%) [36] could be due to varying cultural emphasis on nutrition practices, disparities in healthcare system support for pregnant women and differences in antenatal care protocols regarding food safety protocols. However, these comparisons should be interpreted cautiously due to differences in study design, measurement tools, and population characteristics across studies.

Pregnant women in the current study demonstrated relatively strong knowledge of personal and kitchen hygiene. These findings align with those of a study on pregnant women in Jordan, where a significant number of participants recognized the importance of thoroughly washing their hands before food preparation and after coughing or sneezing while cooking. Similarly, in our study, 65.4% of participants consistently washed their hands after touching any part of their body during food preparation, which is very similar to the Jordanian cohort (57.2%) [36]. Further corroborating this trend, Trepka et al. (2007) found that most participants always washed their hands with soap and running water after changing their baby's diaper or after using the toilet [41]. Trepka et al. (2007) also indicated that a small percentage of participants did not properly clean cutting boards, whereas our study found that 71.2% of participants recognized the importance of cleaning cutting boards during cooking [41]. This suggests potential improvements in hygiene knowledge and regional differences in food safety practices. However, these findings should be interpreted cautiously as self‐reported knowledge may not always reflect actual hygienic behavior.

Similar to our findings, Almanasrah et al. (2022) reported that most participants correctly identified diarrhea and vomiting as common symptoms of food poisoning [36]. Similarly, Al Daour et al. (2022) also reported that most respondents were knowledgeable that vomiting, diarrhea, cramping in the stomach, and pain in the abdomen are symptoms of food poisoning [38]. However, Almanasrah et al. (2022) found that over two‐thirds of the participants were aware that hypertension, low blood sugar, cold, and cough are not symptoms of food poisoning, suggesting that most of our participants were unaware of this information [36]. The difference in awareness between our study and that of Almanasrah et al. (2022) [36] could be attributed to variations in health education exposure, cultural perception of foodborne illness, differences in study populations, or exposure to food safety information. In many areas, symptoms such as vomiting and diarrhea are well known because of their prevalence and severity, making them more commonly discussed in both public health messaging and everyday conversations. However, terms such as hyperglycemia and hypertension are less frequently associated with foodborne illnesses and are not typically mentioned in everyday conversations, which may explain why most participants in our study were less familiar with the fact that these are not symptoms of food poisoning.

The findings from our study, where 85.3% of respondents correctly identified raw, undercooked, or improperly washed food as high‐risk for causing food poisoning, suggest a strong awareness among participants about food safety risks. In contrast, findings from the USA indicate that 48.4% of pregnant women rarely or infrequently consume raw or undercooked meat (such as deli meat or hot dogs), which poses a risk of contracting listeriosis [41]. This shows that while a significant proportion of pregnant women are cautious about consuming high‐risk foods, nearly half may still consume them occasionally, potentially increasing their vulnerability to foodborne illnesses. Approximately, 57% of the respondents in the current study reported using separate cutting boards for meat and vegetables to prevent cross‐contamination, which is lower than the findings from Saudi Arabia (78.5%), where it was recommended that the same cutting board should not be used for both meat and fruits to avoid cross‐contamination [52]. Almanasrah et al. (2022) found that only a small percentage (21.2%) of pregnant women believed that a knife used to chop raw meat should be washed with water, soap, and disinfectant before being used to chop vegetables. This reflects a lower level of knowledge about cross‐contamination among pregnant women in that study than in ours [36].

In the present study, 34.0% of the respondents demonstrated an affirmative answer to the method of food thawing and this finding is in agreement with other studies [53], from China that only 38.2% interviewers knew about the proper way for thawing food. The lack of awareness regarding appropriate thawing techniques may result from low level of training in food safety as well as a poor understanding concerning safe practice. Moreover, Saeed et al. (2021) also found that 54.1% of respondents correctly defrosted frozen meat [18]. It is interesting to note that, in our study, only 43.1% of the participants knew the proper refrigeration temperature compared with 34.5% in Saudi Arabia [52] or with 69.5% in Portugal country [54]. Furthermore, our study found that fewer than half of the subjects (41.1%) were aware that refrigerated left‐over food should be consumed within two to 3 days. This result is in agreement with that of Saudi Arabia, where half of the women gave correct response. Overall, these findings emphasize the necessity for better food safety knowledge, especially regarding thawing, refrigeration and storing.

Around 66.9% of the pregnant women in our study mentioned to have read the instructions provided by manufacturer when using liquid concentrates or ready‐to‐feed products for feeding their babies, which was slightly lower than 74.2% reported among women in Sharjah, United Arab Emirates [18]. This indicates that while most participants are careful with product instruction, there would be some scope for improvement to ensure that all carers understand and follow guidance on safe preparation. In addition, reading food labels is one of the best habits a pregnant mother can contribute in order to aware from protecting their self against foods represented and undermining risk of infection when making them for babies [55]. With regard to the length of room temperature storage, only 50.4% were aware that formula or bottle breast milk should not be left at room temperature for > 2 h, which is an important piece of information because prolonged exposure of feeds to room temperature increases the likelihood of bacterial contamination. However, awareness of this guideline was much higher in Slovenia (83.3%) [11] and Florida, USA (79.4%) [41], indicating that knowledge gaps exist among our population that could place infants a greater risk of foodborne illness. Additionally, almost half (49.3%) of the respondents knew that water should be boiled for at least 2 min before mixing with the formula. Our findings align closely with results from Slovenia (48.9%) [11], but are somewhat lower than those reported in the USA (62.1%) [41]. These results highlight the need for enhanced educational efforts to improve mothers' knowledge of safe infant feeding practices, particularly in low‐income and middle‐income countries.

Our study found that food safety and baby food‐handling knowledge were significantly associated with the age of pregnant women. Interestingly, younger pregnant women, particularly those aged between 18 and 24 years, demonstrated better knowledge in these areas than their older counterparts. However, this finding contradicts findings of other studies which found increasing age groups to possess higher food safety knowledge. For example, older participants (26–34 years) were more likely than younger ones (< 25‐year‐old) to indicate the importance of washing dishes and pots with detergent and hot water after cooking, as well as to clean cutting boards carefully after coming into contact with raw meat in one study [56]. These age‐related differences are consistent with a study among pregnant women, which showed that elderly participants had significantly higher overall food safety knowledge scores compared to their younger counterparts. Such finding was not similar to our finding where younger pregnant women were significantly more knowledgeable about food safety [36]. This discrepancy underscores the relative potential for contexts and demography to influence food safety knowledge between age cohorts.

More food safety and baby foods handling knowledge scores were significantly associated with higher educational level which corroborate earlier investigations [52, 57]. Education and health issues. Higher levels of education are associated with better critical thinking, learning and overall, this can lead to higher awareness regarding health‐related problems. Furthermore, more than half of the pregnant women (53.1%) may have a higher exposure to health information along with formal education and media or even by some healthcare consultants could strengthen their knowledge of food safety. But this can also indicate a disadvantage in the sense that less educated women are possibly less informed about food safety, exposing themselves and their baby to a higher risk. Accordingly, accessible and targeted educational interventions need to be given due consideration for all pregnant women, even those not formally educated, to help maintain their knowledge about baby food‐handling and awareness of the importance of food safety [58, 59].

At the same time, this research showed that urban pregnant women had significantly more food safety and baby food handling knowledge than their rural counterparts. This finding is consistent with those reported by Hamed et al. (2020), who also found that urban participants had better food safety knowledge [60]. The difference in knowledge may be explained by the fact that rural pregnant women do not have enough access to high‐quality education and health service resources. As a result, they cannot consistently absorb new knowledge about food safety or implement food safety regulations into their daily lives [25]. Importantly, our regression analysis revealed that even after taking educational level and other sociodemographic factors into consideration, urban residence alone remained a significant predictor of the level of a father ‘s knowledge about food safety. This suggests that social and demographical factors, such as residential status, exert independent effects on knowledge disparities. This gap in knowledge is worrying: poor knowledge of food safety in rural areas could increase the risk of foodborne illness during pregnancy and have serious health consequences for pregnant women and their fetuses in turn. All mass media platforms, including television, radio and internet‐based channels, can be used as effective tools for popularizing food safety knowledge and empowering pregnant women to make informed choices based on sound information about their food sources [61]. We may have to rely on the convenience of such widely available public health education channels to bridge this knowledge gap between rural and urban areas between mother‐to‐child connections for storage or feeding arrangements, when the woman enters labor.

First trimester versus second/third trimester pregnant females had significantly better food safety or baby food handling knowledge in our study. Food safety knowledge was also affected by the third trimester [16, 36]. One possible explanation is that as pregnancy progresses women may perceive themselves as future caregivers and take steps to prepare for their baby. Such a greater sense of responsibility may inspire them to seek out more health information on safe preparation and handling of food, etc. Additionally, women in the later stages of gestation are more frequently seemingly attend antenatal care visits or prenatal educating seminars within which they may be educated on points pertaining to food hygiene, diet and baby care.

Our study found that the women who were pregnant for two or subsequent pregnancies had much higher levels of knowledge about handling baby foods than those same‐groups who had just had their first child. This suggests that prior experience may provide maternal practitioners with useful knowledge actually about taking care of a child such as healthy eating habits for infants. Parents who have already been through caring for an infant first‐hand, develop practical techniques, learn from their own mistakes whilst things are still fresh in memory and have real‐life problems that form a more comprehensive understanding of the safety preparation and storage of baby food. Nevertheless, it must be pointed out that Asiedu et al. (2021) did not find a significant relationship between the number of deliveries and safety knowledge for the food consumed during pregnancy [16]. This discrepancy may have been due to differences in populations studied, experimental settings or instruments used for measuring results. But the findings of our study underline the importance of providing targeted food safety education for all expectant mothers, but especially those women who are first‐time mothers and so do not yet possess practical experience in any area, including handling food or caring for an infant.

### 4.1 Strengths, Limitations, and Future Research Directions

There were some limitations of this study to be noted. First, since the research was carried out in one district of Kurigram, its application to all pregnant women from different regions of Bangladesh may not be common. Second, perhaps a predetermined sample size was not achieved for this study due to exclusion criteria, limitation of resources and lack of number of participants in the study sites. Third, these being cross‐sectional studies causal inferences cannot be made and instead only associations between the variables can be identified. Fourth, the use of non‐probability sampling may introduce a selection bias, potentially affecting the generalizability of the results. Fifth, the questionnaire and scoring system were not formally validated in the study population prior to data collection, which may introduce measurement bias and affect the accuracy and comparability of the composite knowledge scores. Therefore, interpretations based on overall scores and indices, as well as comparisons involving mean scores, should be made with caution. Finally, the absence of observational validation means that the reported knowledge may not always translate into actual practice. Despite these limitations, this study had several strengths. It provides a comprehensive assessment of food safety and baby food‐handling knowledge among pregnant women in Northern Bangladesh, addressing a critical gap in regional data. The findings were systematically compared with international studies, reinforcing their validity and highlighting global disparities in food safety knowledge. The results align with global research, while emphasizing the need for improved antenatal education, particularly in rural and low‐resource settings. By bridging local and international perspectives, this study supports evidence‐based public health strategies to reduce foodborne disease risk for mothers and infants, making it a valuable resource for policymakers and healthcare providers.

Future research should move beyond single‐site, cross‐sectional surveys to more rigorous and comparative designs. Longitudinal and intervention studies are needed to test whether tailored education during antenatal care can sustainably improve both food safety and baby food‐handling knowledge and actual practices among pregnant women. Cross‐country comparative work would also be valuable. For example, a standardized tool could be applied in Bangladesh and a socioeconomically comparable setting such as Ghana, Jordan or the UAE to examine how cultural context, health systems and communication channels shape the pathways from knowledge to attitudes and practices. Such studies could be theory‐driven, using frameworks like the Health Belief Model or knowledge–attitude–practice models to test how perceived risk, perceived barriers and cues to action (e.g., antenatal counseling, media, community health workers) mediate the translation of knowledge into safe behavior. Finally, future research in Bangladesh should incorporate observational or mixed‐methods approaches to address the well‐documented gap between self‐reported knowledge and real‐life practices in the home, and evaluate the effectiveness and cost‐effectiveness of scalable food safety interventions in low‐resource settings.

## 5 Conclusion

This study concluded that pregnant women in Northern Bangladesh an overall food safety knowledge score of 52.9% and an infant food‐handling knowledge score of 60.9%. However, there are critical gaps in crucial areas such as cross‐contamination prevention, refrigeration temperatures, and thawing techniques. Although participants demonstrated a high level of knowledge regarding sanitation practices, their comprehension of less prevalent foodborne illness symptoms and safe infant feeding practices was inadequate compared to global standards. Factors such as age, monthly family income, higher education, urban residence, advanced pregnancy stage, and previous maternal experience significantly influenced knowledge levels, revealing disparities in access to food safety education. This study underscores the pressing need for targeted antenatal food safety initiatives, particularly for first‐time mothers and those residing in rural areas, to mitigate the risk of foodborne illnesses during pregnancy. The health outcomes of both mothers and infants can be considerably enhanced by incorporating food safety education into routine antenatal care, utilizing mass media campaigns, and providing culturally adapted materials. To evaluate the long‐term effects of these interventions and investigate additional socio‐cultural factors that influence food safety practices, future research should employ longitudinal designs. Healthcare providers and policymakers can promote safer nutritional practices for expectant women and their infants in Bangladesh and other similar settings by addressing these knowledge gaps, thereby enhancing food safety awareness.

## Author Contributions

**Nitai Roy:** conceptualization, methodology, software, investigation, validation, formal analysis, supervision, visualization, project administration, resources, writing – review and editing, writing – original draft. **Sultan Mahmud Imran:** data curation, formal analysis, software, visualization, investigation. **Aysha Siddiky:** visualization, writing – review and editing. **Abdullah Al Adib:** visualization; writing – review and editing. **Md. Mahmud Hasan:** methodology, visualization, writing – review and editing. **A. M. Jubayer:** methodology, visualization, writing – review and editing. **Jasmin Ara Farhana:** visualization, writing – review and editing. **Kamal Krishna Biswas:** visualization, writing – review and editing.

## Funding

The authors have nothing to report.

## Declaration of Generative AI and AI‐assisted Technologies in the Writing Process

The authors (Nitai Roy) acknowledge the use of artificial intelligence (AI) tools (ChatGPT by OpenAI) for assistance with language editing, formatting, and improving the clarity of the manuscript. All content has been reviewed and verified by the authors, who take full responsibility for the final version.

## Conflicts of Interest

The authors declare no conflicts of interest.

## Transparency statement

The Nitai Roy affirms that this manuscript is an honest, accurate, and transparent account of the study being reported; that no important aspects of the study have been omitted; and that any discrepancies from the study as planned (and, if relevant, registered) have been explained.

## Supporting information


**Table S1:** Socio‐demographic characteristics of respondents by study setting.

## Data Availability

The data that support the findings of this study are available on request from the corresponding author. The data are not publicly available due to privacy or ethical restrictions.
