# Supplementary material for: Food Safety and Baby Food Handling Knowledge and Associated Factors Among Pregnant Women in Bangladesh: Findings From a Questionnaire‐Based Cross‐Sectional Survey
Source: Health Sci Rep. 2026 Jun 30;9(7):e72713. doi: 10.1002/hsr2.72713 (PMC13316956; doi:10.1002/hsr2.72713)
Supplement: Supplementary file 1 — Table S1: Socio‐demographic characteristics of respondents by study setting. [file HSR2-9-e72713-s001.docx]

Supplemental Table 1: Socio-demographic characteristics of respondents by study setting

| **Variables** | **Upazila Health Complex (n = 228)** | **Household**  **(n = 113)** | **χ²** | **p-value** |
| --- | --- | --- | --- | --- |
| **Age groups(years)** | | | | |
| 18–24 years | 77 (33.8) | 19 (16.8) | 29.259 | <0.001 |
| 25–34 years | 134 (58.8) | 63 (55.8) |  |  |
| ≥35 years | 17 (7.5) | 31 (27.4) |  |  |
| **Education of pregnant woman** | | | | |
| No formal education | 7 (3.1) | 33 (29.2) | 79.426 | <0.001 |
| Primary | 29 (12.7) | 33 (29.2) |  |  |
| Secondary | 63 (27.6) | 24 (21.2) |  |  |
| Higher secondary | 72 (31.6) | 10 (8.8) |  |  |
| Honors/Master's or above | 57 (25.0) | 13 (11.5) |  |  |
| **Employment status** | | | | |
| Unemployed | 184 (80.7) | 99 (87.6) | 2.555 | 0.110 |
| Employed | 44 (19.3) | 14 (12.4) |  |  |
| **Family monthly income (BDT)** | | | | |
| <15,000 | 87 (38.2) | 36 (31.9) | 2.607 | 0.272 |
| 15,000–30,000 | 94 (41.2) | 57 (50.4) |  |  |
| >30,000 | 47 (20.6) | 20 (17.7) |  |  |
| **Religion** | | | | |
| Islam | 200 (87.7) | 89 (78.8) | 4.691 | 0.030 |
| Hindu | 28 (12.3) | 24 (21.2) |  |  |
| **Residential status** | | | | |
| Rural | 170 (74.6) | 86 (76.1) | 0.096 | 0.756 |
| Urban | 58 (25.4) | 27 (23.9) |  |  |
| **Number of pregnancies** | | | | |
| First pregnancy | 106 (46.5) | 35 (31.0) | 7.502 | 0.006 |
| Second pregnancy and above | 122 (53.5) | 78 (69.0) |  |  |
| **Planned pregnancy** | | | | |
| No | 22 (9.6) | 14 (12.4) | 0.601 | 0.438 |
| Yes | 206 (90.4) | 99 (87.6) |  |  |
| **Pregnancy trimester** | | | | |
| First trimester | 67 (29.4) | 48 (42.5) | 7.171 | 0.028 |
| Second trimester | 124 (54.4) | 45 (39.8) |  |  |
| Third trimester | 37 (16.2) | 20 (17.7) |  |  |
